# New Appliances and Things Medical

**Published:** 1898-04-09

**Authors:** 


### NEW APPLIANCES AND THINGS MEDICAL.

[We shall be glad to receive, at our Office, 28 & 29, Southampton Street, Strand, London, W.C., from the manufacturers, specimens of all new preparations and appliances which may be brought out from time to time.]

### SANOSE.

A New Albumen Preparation (Messrs. A. and M. Zimmerman, 9, St. Mary-at-Hill, London, E.C.)

This is a new albuminous preparation, containing 80 per cent. of casein and 20 per cent. of albumose, and first invented and manufactured by the German chemist Schering. When Sanose is mixed with water a peculiar milk-like fluid results; in fact, it is an emulsion with similar physical and chemical properties. Being a tasteless, nutritious, and easily digested food, it offers great possibilities of providing in combination with selected carbohydrates and fats—an ideal food for invalids whose digestive powers are at fault. In cases of typhoid and marasmus in infants it would appear to be particularly suitable.

### MOSQUERA FLUID BEEF JELLY.

(Mosquera Julia Food Company, New York and Detroit, U.S.A. Parke, Davis, and Co., 21, North Audley Street, London, W.)

This new preparation of beef is as simple in constitution as it is effective in results. It contains in a most concentrated form the nutritive constituents of beef, but differs from many other concentrated bouillons and extracts in that there is absent from it that high percentage of salts and meat extractives which affords the others a false impression of strength. Mosquera Fluid Beef Jelly is unseasoned, and hence, according to the discretion of the consumer, it may be flavoured or not by the addition of condiments. It is a highly valuable nitrogenous food for invalids, concentrated, reliable, and nutritious.

### ASEPTOLIN-EDSON.

(Aseptic Chemical Company, 108, Fulton Street, New York. Agents: Fassett and Johnson, 32, Snow Hill, London.)

This is a new fluid designed for hypodermic or rectal injection in cases of septic poisoning, in malaria, tuberculosis, diphtheria, influenza, and like disorders. It is a solution containing about 3 per cent. of absolute phenol and 0·01 per cent. of a new pilocarpin salt (pilocarpin-phenyl-hydroxide), with a formula $C_{11}H_{16}N_2O_2 OHC_6H_5$. It is claimed for this new antiseptic preparation that when injected into the system the natural power of the blood for resisting and destroying noxious germs are greatly increased.

### OLD SCOTCH CREAM WHISKY.

(R. H. Thomson and Co., Leith and London.)

We have recently made trial of the above Scotch whisky. We find it exceedingly mellow and well matured, and altogether a high grade spirit for general or invalid use. Owing to the care which is taken both in the manufacture and in the maturing of this brand of whisky we highly recommend it to the notice of our readers, feeling assured that a trial of it will confirm the opinion which we have ourselves formed.

### PATENT TYPHOID SAFETY PAN.

(Winton Pottery Company, Limited, Stoke-on-Trent.)

This is an admirable pan for a night commode. It has a lip at one side, by which its contents can be poured out n a much more cleanly manner than is possible where there is a nick in the rim to contain water. At the same time the lid can be made quite as air-tight as those which are provided with a water seal. The fixing of the lid is, in fact, the special feature in this safety pan. It is contrived after the manner of a tobacco jar. Above the lid is a branched metal plate, the three points of which are placed in spiral grooves in the sides of the pan. By a slight twist the indiarubber edge of the lid is brought in contact with the pan, and by a turn of a thumbscrew all is made secure. Our only objection is to the name which has been given to this invention. Typhoid patients do not usually sit up on night commodes.

